# Chimeric Antigen Receptors Directed at Mutant KRAS Exhibit an Inverse Relationship Between Functional Potency and Neoantigen Selectivity

**DOI:** 10.1158/2767-9764.CRC-21-0165

**Published:** 2022-01-28

**Authors:** Talar Tokatlian, Grace E. Asuelime, Martin S. Naradikian, Jee-Young Mock, Mark E. Daris, Aaron D. Martin, Dora Toledo Warshaviak, Alexander Kamb, Agnes E. Hamburger

**Affiliations:** 1 Research, A2 Biotherapeutics, Agoura Hills, California.

## Abstract

Neoantigens are among the most intriguing potential immuno-oncology targets because, unlike many cancer targets that are expressed on normal tissues, they are by definition restricted to cancer cells. Medicines directed at common neoantigens such as mutant KRAS are especially interesting because they may offer the convenience and cost of an off-the-shelf therapy. However, all common KRAS mutations produce proteins that differ from the wild type at a single amino acid, creating challenges for molecular discrimination. We have undertaken an effort to optimize single-chain variable fragments (scFv) against peptide/major histocompatibility antigen complexes composed of HLA-A*11 and either G12V- or G12D-mutant KRAS peptides. These scFvs could in principle be used in chimeric antigen receptor (CAR) T-cell therapies for selected patients whose tumors bear either of these mutations. Here we show that optimization of such CARs involves a trade-off between potency and selectivity. We further show that targeting this family without high selectivity engenders risks of cross-reactivity against other members of the G-protein family to which KRAS belongs.

**Significance:**

We report an effort to generate high potency, selective CARs directed at mutant KRAS peptides. Although the heavily optimized CARs maintain high selectivity against wild-type KRAS, they lose selectivity against other KRAS-related peptides derived from human proteins. To our knowledge, this work is the first to examine the trade-off between potency and selectivity with regard to KRAS pMHC-directed CARs, illustrating the challenge to achieve both sufficient potency and high selectivity.

## Introduction

Since the discovery of KRAS as a mutated oncogene nearly 40 years ago, cancer therapeutics that exploit this opportunity to distinguish selected tumors based on a functional dependency or biochemical difference have been much sought after (1). While small molecules have had success in targeting specific KRAS point mutations (2–4), immunologic interventions have been limited. Indeed, mutant KRAS presents significant obstacles: (i) it is an intracellular protein and thus requires presentation on highly polymorphic MHC molecules; (ii) KRAS-reactive receptors need sufficient sensitivity to recognize the low level of expression of most pMHCs, while maintaining specificity for a few distinctive atoms (e.g., G12V); and (iii) KRAS is a member of a large family of G-proteins that contain numerous homologous sequences surrounding the commonly mutated sites.

Cell therapy utilizing T-cell receptors (TCR) or chimeric antigen receptors (CAR) offer a potential solution to some of these problems. Screening patients or immunization of HLA-transgenic mice with common MHC class I alleles have yielded some selective TCRs (5–11), although only a few have progressed into the clinic. mAbs provide an alternative route to mutant KRAS-targeted receptors for immunotherapy. The capacity of mAbs to recognize not only single amino acid substitutions (12) but also specific pMHC determinants has long been appreciated (13, 14). To that end, recent library display methods have been used to identify mAbs and single-chain variable fragments (scFv) that bind pMHCs differentially based on single amino acid differences in the peptide portion of the target complex (15). Skora and colleagues reported an A*11/KRAS/G12D mAb with good discrimination against wild type (WT; ref. 16). However, these mAbs (and scFvs) obtained via phage display exhibited limited affinity (double-digit nanomolar Kd) for their pMHC targets, even after optimization of their binding elements.

Here we describe an effort to achieve higher functional potency for KRAS mutant–selective binders. We focus on isolation of scFvs that mediate selective activity against A*11/KRAS G12V as CAR-Ts. Combination of enrichment for binders and secondary functional screens yielded CARs selective for A*11/G12V. Two additional optimization cycles generated CARs with enhanced potency, but reduced specificity against other A*11 pMHCs. We conclude that there is a gap between the dual properties of potency and selectivity that may be difficult to breach for CARs directed at KRAS-mutant pMHC targets.

## Materials and Methods

### Cell Lines, Lentivirus, and Peptides

Jurkat NFAT-Firefly-Luciferase cells were purchased from BPS Bioscience. T2, HeLa, HuCCT1, PANC1, SHP77, SW527, and A375 cells were purchased from ATCC between 2018 and 2019. COLO 668 cells were purchased from Sigma in 2019. All cell lines were initially tested for *Mycoplasma* using the commercially available MycoAlert *Mycoplasma* Detection Kit from Lonza and used for less than 30 passages. Furthermore, cell lines were authenticated by HLA typing at The Sequencing Center to confirm reported HLA alleles. Available data on *in vitro* growth properties and morphology from ATCC was also used to confirm cell identity. HLA-A, -B, and -C alleles were knocked out via CRISPR/Cas9 gene editing from T2 cells, which were subsequently transduced with lentivirus encoding HLA-A*11:01 at a multiplicity of infection (MOI) = 5, and flow sorted for HLA class I expression using W6/32 antibody. Custom lentiviruses were purchased from Alstem. Peptides, including KRAS G12V 10 mer and 9 mer (VVVGAVGVGK, VVGAVGVGK), WT KRAS 10 mer and 9 mer (VVVGAGGVGK, VVGAGGVGK), and all selectivity peptides, were purchased from GenScript.

Peripheral blood mononuclear cells (PBMC) from healthy donors were obtained from AllCells. PBMC collection and donor written informed consent were approved by an Institutional Review Board, with strict oversight. In addition, HIPAA compliance and approved protocols were followed for all work involving human T cells.

### Biochemical Screen for Stable pMHC Complexes

pMHC AlphaScreen was performed as described previously (17). Briefly, refolding reactions were carried out with beta-2 microglobulin (B2M; 0877095-CF, MP Biomedicals), purified alpha subunit (0.3 μmol/L A*01:01 + 0.6 μmol/L B2M, 1 μmol/L A*02:01 + 2 μmol/L B2m, 0.1 μmol/L A*03:01 + 0.2 μmol/L B2m, or 0.1 μmol/L A*11:01 + 0.2 μmol/L B2m) and four concentrations of KRAS peptides [50 μmol/L, 5 μmol/L, 0.05 μmol/L, or 0.005 μmol/L (Genscript)]. The mixtures were incubated at 37°C for 18 hours then transferred to a 384-well Proxiplate (6008289, PerkinElmer). A 1:1 mixture of streptavidin-conjugated donor beads (6760002B, PerkinElmer) and acceptor beads (6762002, Perkin Elmer) conjugated with 1 mg/mL W6/32 antibody (BE0079, Bioxell) was added at a final total bead concentration of 20 μg/mL. Signal was measured via EnSpire 2300 (PerkinElmer) after a 1-hour incubation at room temperature.

### Mass Spectroscopic Analysis of Peptides

Mass spectrometry experiments were conducted by Caprion Biosciences on human cell lines as described previously (15). The mass spectrometry proteomics data will be deposited to the ProteomeXchange Consortium through the PRIDE partner repository.

### Binder Generation Using Mammalian Cell–Based *In Vitro* Display

Use of HuTARG libraries to obtain pMHC-specific binders has been described previously (15, 17). Briefly, pMHC probes were generated as described previously (18). The library was enriched using a BD FACSAria cell sorter for cells displaying IgGs that bind specifically to mutant KRAS pMHC tetramer probes (both 10 mer and 9 mer G12V/A*11:01), but not to WT KRAS tetramer pMHC probes. Multiple enrichment rounds were performed to increase on-target and decrease off-target binding. In the final round, both on-target and off-target binding cells were collected. RNA was extracted from these pools and reverse transcribed into cDNA. PCR fragments containing the complementarity-determining region (CDR) regions were generated using the cDNAs as template, followed by targeted next-generation sequencing (NGS) to determine the frequency of each binder with a unique CDR region. The degree of enrichment/depletion was determined by comparing the output and input NGS counts.

Target-specific binders from the primary libraries were used to generate optimization libraries to further improve on-target sensitivity. Optimization libraries were constructed by diversification of CDR-1, CDR-2, or CDR-3 light chains or heavy chains of parent binders by *in vitro* RAG-mediated V(D)J recombination. Enrichment and NGS analysis to identify optimized binders were performed as described above.

### Molecular Cloning of CAR Constructs

CAR constructs were created by fusing an scFv LBD to a hinge, a transmembrane domain (TM) and an intracellular signaling domain (ICD). The hinge was derived from CD8, the TM from CD28, and the ICD from CD28, 4–1BB and CD3ζ. Gene segments were combined using Golden Gate cloning and inserted downstream of a human EF1alpha promoter contained in a lentivirus expression plasmid.

### Jurkat NFAT Functional Assay

Jurkat NFAT activation functional assays were conducted as described previously (15, 17). Briefly, Jurkat cells were transfected with TCR or CAR constructs using standard protocols for the Neon transfection system (Invitrogen, MPK5000). T2 or other HLA-A*11(+) cells were concurrently loaded with serially diluted KRAS peptides, ranging from approximately 20 pmol/L to 100 μmol/L peptide, including a control at 0 μmol/L peptide (E_min_). Peptide-loaded HLA-A*11 cells were incubated overnight at 37°C in 384-well plates (Corning, 3570), plated at 10,000 cells per well. A total of 16–18 hours posttransfection, Jurkat cells (10,000 cells/well) were added to the peptide-loaded HLA-A*11 cells to a final volume of 30 μL. After a 6-hour incubation at 37°C, the One-Step Luciferase assay system (BPS Bioscience, 60690) was used to determine luminescence intensity using a Tecan Infinite M1000.

For near off-target selectivity screening, individual peptides were loaded onto T2-A*11 cells at 100 μmol/L peptide and function was normalized to G12V 10-mer peptide. Selectivity peptide identification was done exactly as described previously (17) and in the Supplementary Data.

To assess TCR or CAR expression by flow cytometry, transfected Jurkat cells were stained using protein L (Thermo Fisher Scientific, 29997) or anti-mouse TCRβ (BioLegend, 109208). Binding to KRAS mutant or WT pMHC tetramers was similarly assessed following a 60-minute incubation with 10 μg/mL pMHC tetramer either individually or pooled (co-stain).

### Primary T-Cell Assays

PBMCs from healthy donors, obtained from AllCells, were cultured and transduced with KRAS TCR or CAR constructs using custom lentivirus (Alstem) as described previously (15, 17). In the absence of enrichment, primary T cells were stained for TCR or CAR expression and pMHC probe binding similarly as described above for Jurkat cells. CD4 and CD8 T-cell populations were also characterized by flow cytometry. A total of 15 days posttransduction transduced primary T cells were enriched via positive selection by staining with protein L-biotin/streptavidin-PE or mTCRβ-PE followed by anti-PE microbeads (Miltenyi Biotec, 130-048-801) and subsequent separation through LS columns (Miltenyi Biotec). T cells were further expanded for 5 days and then cocultured with 25,000 target cells at an effector:target = 1:1 for 24 hours with peptide-pulsed T2 HLA-A*11, endogenous KRAS G12V(+) SHP77 ± HLA-A*11, or endogenous KRAS G12D(+)HLA-A*11(+) PANC-1. Secreted IFNγ in the culture was assessed by ELISA (Thermo Fisher Scientific, 88-7316-88).

### Data Availability

The data generated in this study are available within the article and its Supplementary Data files.

## Results

### Isolated KRAS G12V pMHC mAbs Demonstrate Selectivity Over WT KRAS

We focused on HLA-A*11 and G12V/D because of previous work that characterized TCRs derived from A*11 transgenic mice immunized with A*11/G12V/D tetramers (11). These TCRs are weak or inactive against native cell lines unless loaded with peptide or engineered to overexpress A*11. Therefore, we first sought to gain confidence that unmanipulated KRAS G12V/D(+)/HLA-A*11(+) cell lines display the KRAS-mutant peptides. We used pMHC immunoprecipitation and mass spectrometry to search for peptide spectra in four such cell lines (Table 1). We identified spectra for only one KRAS 10-mer peptide (KRAS_7–16_, G12V) in the KRAS G12V(+)HLA-A*11(+) COLO 688 cell line. No other KRAS peptides, WT or mutant, were detected in the remaining lines, although various WT KRAS peptides were detected in some A*02(+) cell lines tested. We also searched for other mutant KRAS peptides for which we had obtained biochemical evidence of complex formation with common HLA-A alleles [G12V/HLA-A*02 (Supplementary Fig. S1)] in one additional mutant cell line (SW527), although none were identified. This result is consistent with the low levels of most pMHCs on the cell surface (19). However, we also note that mass spectrometry is known to have false-negative results in the range of pMHC abundance (20). The discrepancy between total KRAS protein and apparent presentation is well documented (21, 22). Because only approximately 1% of pMHCs have abundance of at least 2,000 molecules/cell, we assume A*11/G12V, though detectable by mass spectrometry, is considerably less abundant (19). Thus, potent receptors are likely required to mediate effective killing by T cells at physiologic levels of mutant KRAS.

**TABLE 1:** Mass spectrometry confirms display of 10-mer G12V KRAS neoantigen peptides in COLO 668 cell line.

Cell line (# of cells)	KRAS mutation status	HLA-A	HLA-A expression (RPKM)	KRAS expression (RPKM)	Wild-type KRAS peptide identified	KRAS neoantigen identified
A375 (3.5e8)	WT	A*01:01 A*02:01	64	5	KSFEDIHHY_88–96_	Na
HuCCT1 (5e8)	G12D	A*11:01 A*11:01	129	1	—	—
PANC-1 (5e8)	G12D	A*02:01 A*11:01	149	5	—	—
COLO 668 (5e8)	G12V	A*01:01 A*11:01	125	3.8	—	—
COLO 668 (1e9)	G12V	A*01:01 A*11:01	125	3.8	—	VVVGA**V**GVGK
SW527* (5e8)	G12V	A*02:01 A*24:02	104*	52*	QYMRTGEGF_70–78_	—
SHP77^#^	G12V	A*02:01 A*24:02	59	6	Nt	Nt

NOTE: Summary of mass spectrometry and gene expression data [Cancer Cell Line Encyclopedia and TRON(*) databases] are shown. The 10-mer G12V KRAS neoantigen was detected in COLO 668 cells when 1e9 cells were subjected to pMHC purification and peptide extraction. SHP77^#^ cell line was not included in the mass spectrometry study.

Abbreviation: na, not applicable; nt, not tested.

On the basis of these biochemical and mass spectrometry findings and the literature, we limited further efforts to G12V peptides complexed with A*11. We screened a library of random antibodies generated by induced RAG1-mediated rearrangement of heavy (H) chain gene segments in HEK293 cells, paired with a common kappa light (L) chain (15, 17). We used A*11 complexed with KRAS G12V peptides in tetrameric form as a labeled probe, followed by counter screens with pMHCs composed of A*11/WT KRAS peptides. Because the G12V-specific TCR identified previously reacted with a 9-mer KRAS G12V peptide, and we only observed a 10-mer peptide via mass spectrometry, we screened with a mixture of both 9- and 10-mer peptides in the tetramer probe. We found good enrichment of A*11/KRAS binders after three rounds of screening (Supplementary Fig. S2A). Binders were sorted and submitted for NGS to identify unique sequences for subsequent functional assessment in Jurkat cells.

**FIGURE 1:**
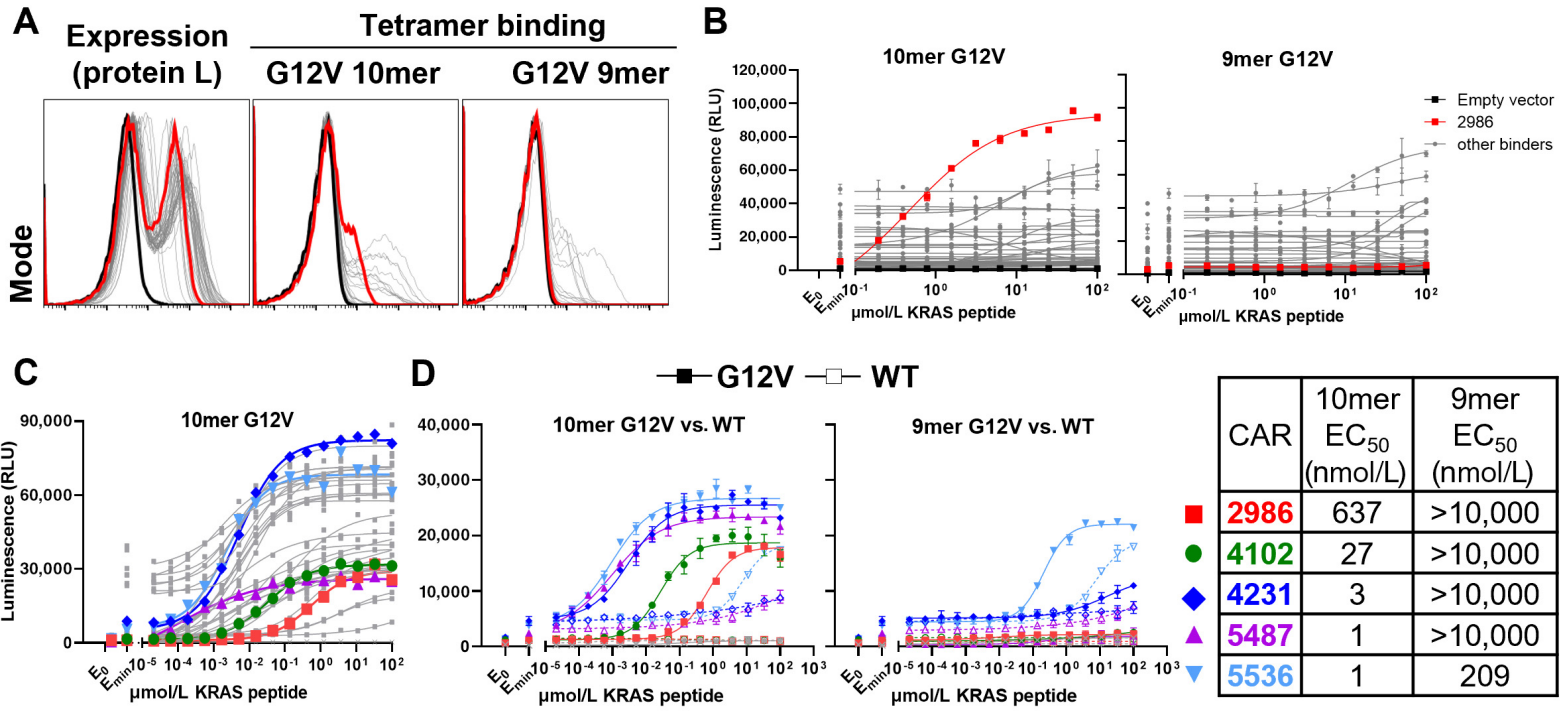
Mutant KRAS–selective binders confer specific responses as CAR constructs in Jurkat cells. **A,** Primary binder expression assessed by binding to protein L or pMHC tetramers. The lead primary binder, 2986, is highlighted in red; see Supplementary Fig. S2A and Materials and Methods for screening details. **B,** Functional characterization of primary binders in Jurkat cell assays. T2-A*11 cells were loaded with serially diluted G12V 10-mer or 9-mer peptides overnight and subsequently cocultured with Jurkat-NFAT-luciferase cells transiently transfected with indicated CARs for 6 hours. **C,** Functional characterization of H chain– and L chain–optimized binders derived from the lead 2986 (red). Top four CARs show improved potency over the parental CAR. **D,** Lead optimized CARs were assessed for activation by 10-mer and 9-mer G12V peptides (closed) versus 10-mer and 9-mer WT peptides (open).

We next tested these binders as scFvs in CAR constructs; a total of 54 idiotypes that were enriched were tested. We first confirmed that the individual scFv CAR constructs were expressed on the surface of Jurkat cells, although not all bound strongly to pMHC tetramers (Fig. 1A). To test their function, we generated an A*11-positive variant T2 cell line and utilized this line in all subsequent Jurkat:T2 peptide-loading assays. The CAR constructs displayed a range of reactivities and backgrounds. We chose one (2986) for further study based on its potency and selectivity over WT KRAS peptide. This CAR responded strongly to KRAS G12V 10-mer peptide, but not to 9-mer peptide (Fig. 1B). *In toto*, these results affirm the use of the mammalian cell line screening strategy for the identification of functional binders that appeared to be pMHC specific.

### Optimization of KRAS Binders Results in Higher Sensitivity, but Reduced Selectivity

Because the most potent, selective CARs derived from the primary (unoptimized) binder screen were only approximately 1 μmol/L in the T2 assay, well below the best subnanomolar TCRs and CARs (17), we attempted to improve the sensitivity of 2986 by CDR optimization following the schema of Supplementary Fig. S2B. Briefly, we independently optimized the H- and L-chain CDRs in parallel, keeping one chain constant and varying the CDRs for the other in scFv constructs. We then paired together the best binders (defined by both EC_50_ sensitivity and low tonic signaling) from the H- and L-chain optimization campaigns resulting in 19 distinct, fully optimized scFvs (top binders described in Supplementary Table S1). These were tested as CARs in Jurkat:T2 functional assays for sensitivity and selectivity against WT peptide (Fig. 1C and D). Although some were less sensitive than the 2986 parent, many displayed dramatically lower EC_50_s. Four of these CARs (4102, 4231, 5487, 5536) from each optimization stage, were selected for further study based on their potency and low background. They ranged from 30–500× more sensitive EC_50_s in the Jurkat:T2 peptide-loading assay using KRAS G12V 10 mers. One CAR (5536) also demonstrated reactivity toward the G12V 9-mer peptide.

**FIGURE 2:**
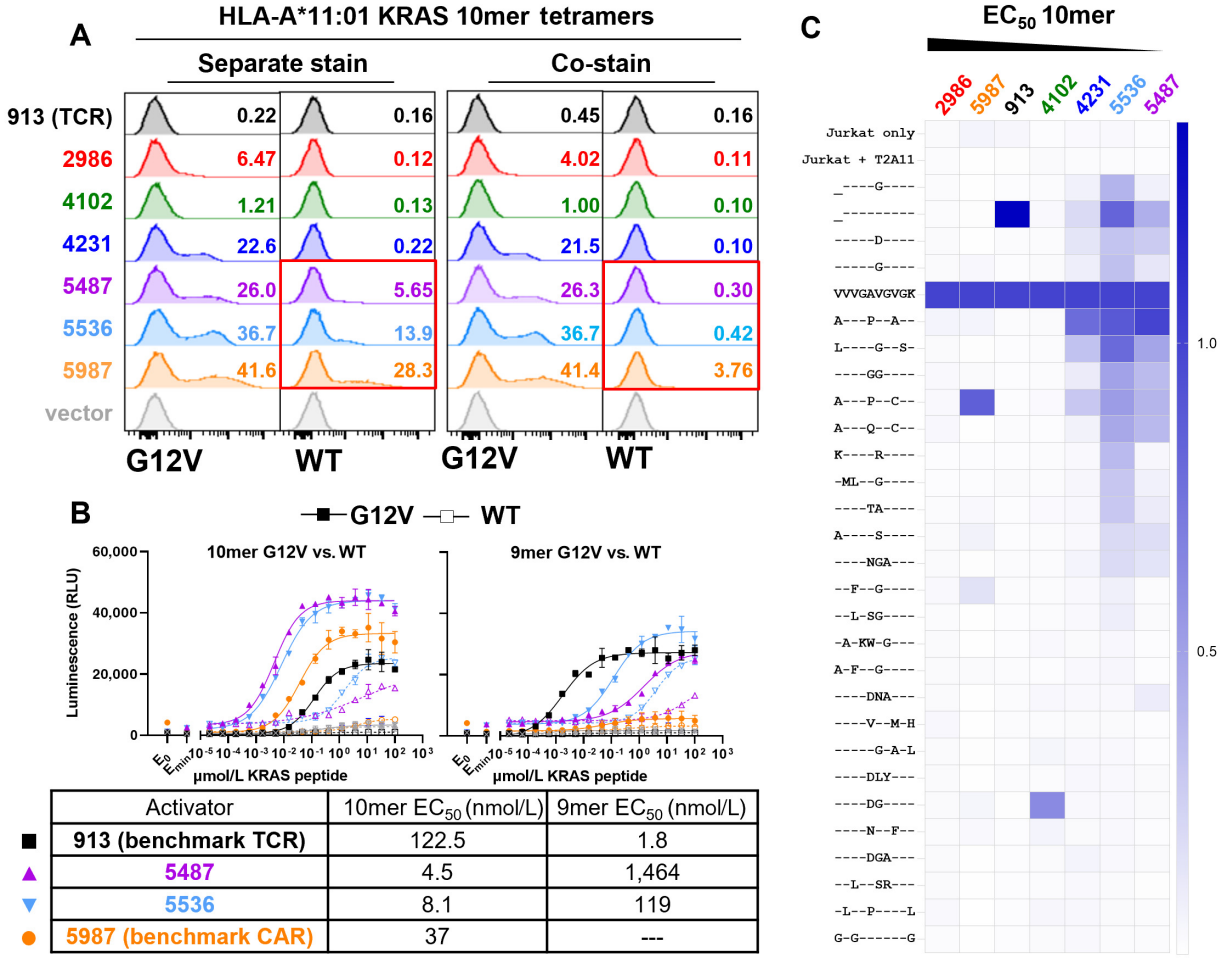
Comparison of lead CARs with benchmark TCR and CAR show cross-reactivity of more potent CARs. **A,** Transiently transfected Jurkat cells were stained with pMHC tetramers either separately (left two panels) or concomitantly (right two panels). **B,** Functional activity of CARs against 10-mer and 9-mer G12V or WT peptides in Jurkat:T2 assays with benchmark comparisons. **C,** Heatmap of functional cross-reactivity against a panel of near-off-target peptides loaded in T2-A*11 cells with peptide at 100 μmol/L. Constructs are plotted in order of increasing potency and normalized to 10-mer G12V reactivity.

We next examined in detail the binding and functional selectivity of the four optimized CARs and compared them with a benchmark KRAS TCR [913 (11)] and a CAR derived from a KRAS peptide-targeted scFv described previously (5987; Patent: US20200079854A1). Three of the four optimized CARs showed detectable binding to the KRAS G12V 10-mer pMHC tetramers, as did the benchmark CAR (Fig. 2A). The parental CAR showed weak binding, and the TCR exhibited none despite robust expression (Supplementary Fig. S3). Two of the four CARs and the benchmark CAR also displayed some binding to WT KRAS tetramer; however, this cross-reactivity disappeared when the mutant and WT tetramers were mixed, presumably due to competition for receptor. In Jurkat:T2 functional assays, one of the four optimized CARs (5536) also displayed significant cross-reactivity to both KRAS 9 mer and 10 mer (Fig. 2B). Nonetheless, the selectivity ratio between mutant and WT peptides was still high for certain constructs, up to 1,000×.

We sought to examine selectivity of the constructs more broadly, especially because the human genome encodes many peptide sequences homologous to the region of KRAS that spans position 12. These sequences are a possible source of cross-reactivity and could pose a safety risk for CARs and TCRs selected to bind mutant KRAS pMHCs. It is known that TCRs can display dangerous cross-reactivity to displayed peptides that differ by as many as four residues (23). We therefore explored possible cross-reactivity of peptides, identified as described previously (17), of genes expressed in adult human tissue (Supplementary Fig. S4). We compared the functional response of the optimized CARs and benchmarks against 26 10-mer peptides, including WT and G12D, differing in sequence from KRAS G12V at 1–4 positions. 9-mer G12V and WT peptides were also included for reference. All constructs except for the TCR and 2986 revealed some cross-reactivity in peptide-loading assays at 100 μmol/L peptide concentration (Fig. 2C). Strikingly, the most potent CARs were the most cross-reactive. Whereas the parental CAR (2986) did not respond to any homologous peptides, the most sensitive CARs (5536, 5487), with EC_50_s in Jurkat:T2 assays comparable with the benchmark TCR, yielded significant signals for at least 10 of the 26 peptides tested. Importantly, we performed functional peptide-titration tests in TAP-sufficient cell lines and found that overt cross-reactivity correlated with A*11 expression which was masked when using T2 cells (Supplementary Fig. S5A and S5B). In summary, though all the optimized CARs exhibited functional activity against A*11/G12V greater than the benchmark TCR isolated *in vivo*, none achieved this high potency without sacrificing some selectivity.

### KRAS-Specific CARs and TCR Lack Sufficient Potency to Recognize Native G12V pMHCs

**FIGURE 3:**
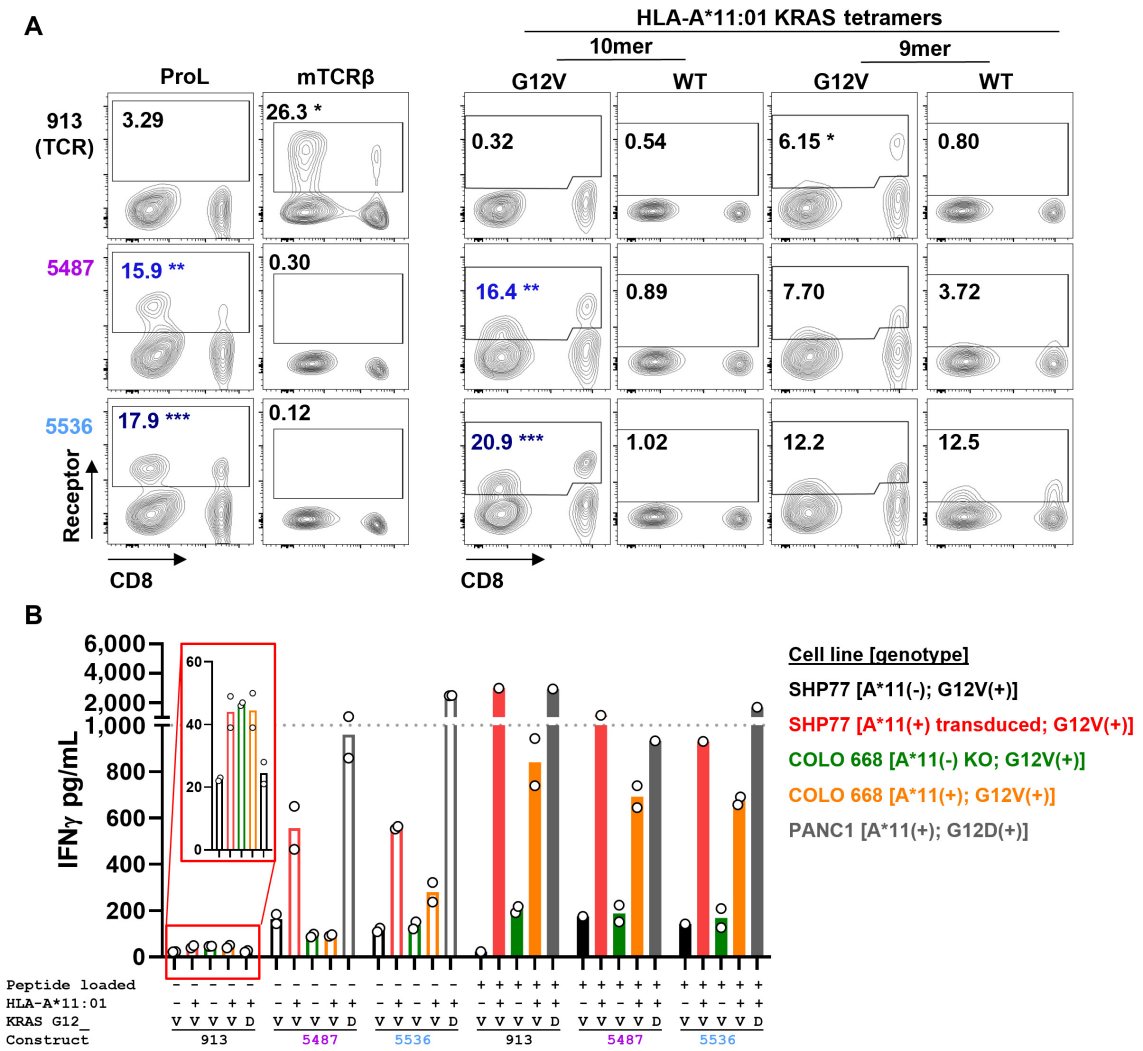
Primary T-cell assays show reactivity against endogenous pMHC by IFNγ secretion. **A,** Primary T cells were transduced with indicated constructs and stained for receptor expression and tetramer binding. **B,** IFNγ levels measured from the supernatant of cocultures with transduced T cells enriched by binding to mTCRβ or protein L (ProL) to a final proportion of >90% of T cells. Cocultures with various KRAS-mutant cell lines were performed to assess the capacity of each construct to recognize endogenously processed and presented KRAS G12V-mutant peptide (open bars) or loaded 10-mer KRAS G12V peptide at 1 μmol/L (closed bars). Gray dotted line indicates limit of detection.

The ultimate *in vitro* test of receptor sensitivity involves primary T cells as effectors and target cells with native expression of KRAS and A*11. We therefore conducted functional studies for specificity/sensitivity with primary T cells using a variety of cells lines with and without peptide loading. Several of these lines are naturally A*11(+)/KRAS G12D or V(+) with endogenous expression of these genes (Table 1). The receptors expressed well in primary T cells (Fig. 3A). Binding studies with pMHC tetramers suggested some dependence of the TCR on CD8 coreceptor expression, while the tetramers bound equally to CAR-positive T cells regardless of their CD4/8 status. All constructs mediated strong response by target cells in T cells measured by IFNγ secretion when the targets were A*11(+) and loaded with exogenous peptide (Fig. 3B, filled bars). The optimized CARs (5487 and 5536) were also strongly reactive to target cells without peptide if the targets expressed A*11 either naturally or via overexpression. However, this reactivity appeared to be independent of KRAS G12V peptide as it was observed in the G12D-mutant cell line, PANC1. Furthermore, even cell lines lacking HLA-A*11:01 expression (SHP77 WT) exhibited some degree of activation suggesting that cross-reactivity was broader than HLA-A*11:01 alone. Because no constructs responded strongly to KRAS G12D 9-mer or 10-mer peptide-loaded T2 cells, this result implies cross-reactivity to pMHC complexes present normally on the epithelial cell lines, but not present on TAP-deficient T2 cells. Only the TCR displayed high target selectivity in these functional assays; however, it responded weakly to a native cell line (COLO 668) expressing KRAS G12V protein as reported previously (11). We attribute this result to the fact that the TCR preferentially recognizes the KRAS G12V 9-mer, but only the 10-mer was detected by mass spectrometry (Table 1). The combination of some presentation of the 9-mer pMHC to which the TCR is especially sensitive and the benefit accrued to the TCR by CD8 expression on primary CTLs may partly explain the detectable, albeit weak, response. Furthermore, Wang and colleagues (11) reported variable IFNγ secretion (from less than 100 pg/mL to nearly 10,000 pg/mL) depending on the relative levels of endogenous KRAS mRNA of each target cell. COLO 668 and SHP77 may indeed be on the lower end of the spectrum of what was reported. Moreover, our HLA-A CRISPR knockout (KO) COLO 668 line is incomplete (Supplementary Fig. S5C). While IFNγ secretion is apparently different between WT and KO, peptide-pulsed COLO 668, we find comparable IFNγ levels for the TCR without exogenous peptide. We argue that the TCR is sufficiently sensitive to recognize these few residual WT cells leading to comparable IFNγ signal between WT and KO. To summarize, none of the constructs studied here—either the optimized CARs, the phage display–derived benchmark CAR, or the TCR—had sufficient sensitivity/selectivity to mediate potent, specific recognition of target cell lines with native expression of A*11 and mutant KRAS.

## Discussion

Individually characterized TCRs and mAbs with high potency and selectivity against mutant KRAS to mediate clinical activity have been hard to come by. In the case of native TCRs isolated *in vivo*, such endogenous receptors appear to be rare, and consequently seldom emerge from broad screening efforts of cancer patients’ TCR repertoires. In this study, we sought to optimize binders from an *in vitro* repertoire of randomly rearranged mAbs, using low-throughput functional assays to address the issue that binding is necessary but not sufficient for potent activity (18). This approach may provide some benefit over optimization based purely on binding. Nonetheless, it did not produce CARs with sufficient functional activity to mediate strong cytotoxicity in unmodified mutant KRAS-positive tumor cell lines. Comparison of different target cell lines as stimuli suggests that the loss of selectivity at the functional level after optimization likely can be traced to non-KRAS peptides displayed in complex with A*11 protein. It is not due to interactions with the A*11 molecule alone. The experiments with closely related peptides derived from G-protein paralogs in the genome support the view that some of these peptides in complex with A*11, many of which are expressed in epithelial cells, may activate the receptors differentially if displayed at sufficient levels on the cell surface. This finding underscores the risks associated with optimization of binding of the receptors used in cell therapies.

The possibilities for improving the potency/selectivity of CARs directed at mutant KRAS pMHCs include: (i) higher diversity of the initial and/or optimization binder libraries (24); (ii) more complex, diverse counterscreens during binder selection; (iii) structure-based design (25); and (iv) higher throughput functional screens. TCR-Ts remain an attractive option if: (i) naturally occurring TCRs of sufficient potency can be identified; or, (ii) TCRs can be optimized to increase potency without sacrificing selectivity. Indeed, TCR optimization via phage display has been associated with at least one case of off-target reactivity that caused fatal toxicity in the clinic (23). We conclude that generation of mutant KRAS–selective TCRs and CARs may benefit from high-throughput functional assays to optimize both features—potency and selectivity—in concert.

## Supplementary Material

Supplementary Data S.Figures 1-5 and S.Table 1
