# Supplementary material for: Chimeric Antigen Receptors Directed at Mutant KRAS Exhibit an Inverse Relationship Between Functional Potency and Neoantigen Selectivity
Source: Cancer Res Commun. 2022 Jan 28;2(1):58–65. doi: 10.1158/2767-9764.CRC-21-0165 (PMC9973398; doi:10.1158/2767-9764.CRC-21-0165)

Supplementary materials

Chimeric Antigen Receptors Directed at Mutant KRAS exhibit an inverse relationship between functional potency and neoantigen selectivity

Talar Tokatlian, Grace E Asuelime, Martin S Naradikian, Jee-Young Mock, Mark E Daris, Aaron D Martin, Dora Toledo Warshaviak, Alexander Kamb, Agnes E Hamburger

Supplementary Figure 1.

Identification of HLA-A allele complexes with KRAS peptides. (**A**) Alphascreen™ to identify KRAS neoantigen pMHC complexes. KRAS G12V and G12D peptides were screened against four frequent HLA-A alleles. (**B**) pMHC generation and characterization of pMHC complexes, including positive hits. Experimental methods are described in Wang et. al. 2021. NCI TCR benchmarks are indicated ([1]). nt = not tested.

A

Primary screen

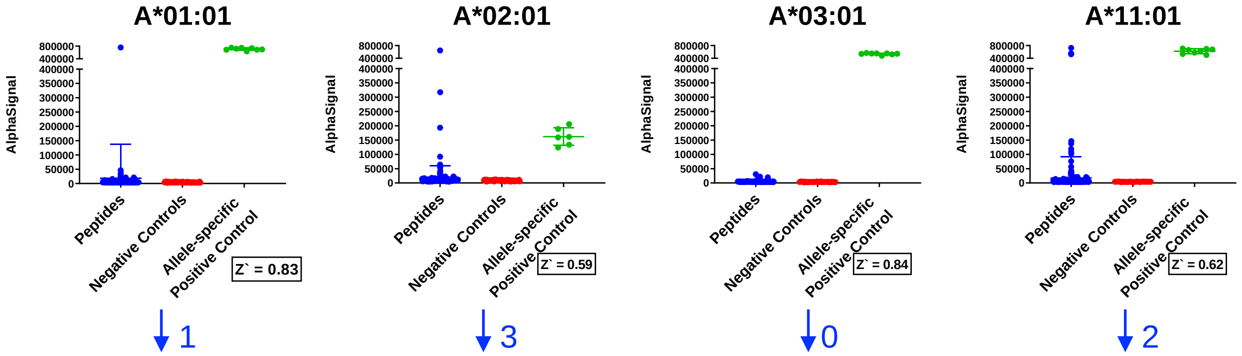

Hit confirmation

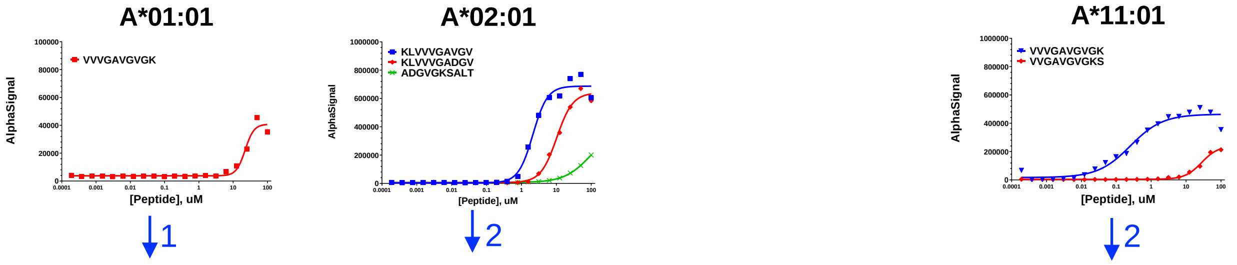

B

KRAS Mutation	Peptide length	Peptide Sequence	Allele	AlphaScreen	Tm (°C)	$T_{1/2}$ (min, 37°C)
G12V	10	VVVGA**V**GVGK	A*01:01	+	Failed refold	nt
	10	KLVVVGA**V**GV	A*02:01	+	54.6	51.6
	9	VVGA**V**GVGK	A*03:01	-	48.9	43.8
	10	VVVGA**V**GVGK	A*03:01	-	50.7	79.8
	9	VVGA**V**GVGK[1]	A*11:01	-	59.3	56.4
	10	VVVGA**V**GVGK[1]	A*11:01	+	60.5	55.2
	10	VVGA**V**GVGKS	A*11:01	+	nt	nt
G12D	10	KLVVVGA**D**GV	A*02:01	+	50.3	18.6
	10	A**D**GVGKSALT	A*02:01	low	nt	nt
	9	VVGA**D**GVGK	A*03:01	-	41.8	nt
	10	VVVGA**D**GVGK	A*03:01	-	45.5	28.8
	9	VVGA**D**GVGK	A*11:01	-	50.9	21.0
	10	VVVGA**D**GVGK[1]	A*11:01	-	58.3	43.8

Supplementary Figure 2.

Isolation of selective KRAS G12V neoantigen binders. (**A**) To identify potent and selective KRAS-directed ligand binding domains (LBDs) that function as CARs, a mammalian surface display library encoding IgG antibodies were enriched through multiple rounds of on- and off-target cell sorting. In the final round, on-target and off-target binders were separately isolated and subjected to next-generation sequencing (NGS) to determine the frequency of each CDR. By comparing the input and output NGS counts, sequences that were enriched only in the on-target binding population, and not for off-target binding, were selected and cloned for further characterization. (**B**) Binder optimization schema. The lead primary binder was subjected to H and L chain optimization in scFv format through additional cell sorting. Optimized L and H chains were mixed-and-matched in additional CAR constructs.

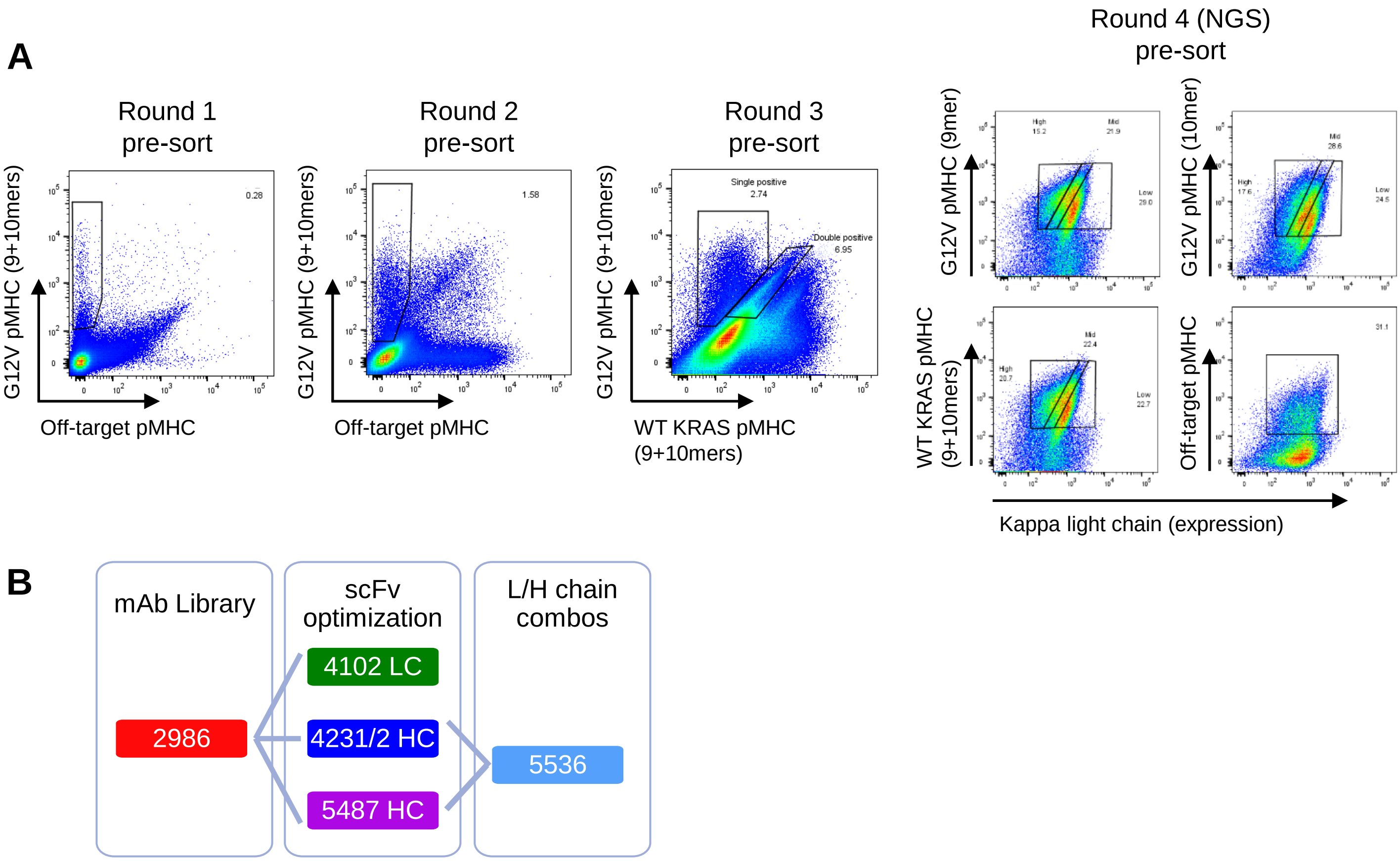

Supplementary Table 1.

CDR sequences from primary, optimized and benchmark binders. Reported benchmark CDR regions are underlined.

Construct	HCDR1	HCDR2	HCDR3	LCDR1	LCDR2	LCDR3
2986 parent	DYAMH	GISWNSGSIGYADSVKG	DIAYDYSDLAYRYLNWFDP	RASQSISSYLN	AASSLQS	QQSYSTPLT
4102 LC opt	DYAMH	GISWNSGSIGYADSVKG	DIAYDYSDLAYRYLNWFDP	RASQSISSYLN	AATAPQDVLQS	QQSYSTPLT
4231 HC opt	DYAMH	GISWNSGRIGYADSVKG	DIAYDYSDLAYRYLNWFDP	RASQSISSYLN	AASSLQS	QQSYSTPLT
4232 HC opt	DYAMH	GISWNSGSIGYADSVKG	DIAYDYSDLAYRYLGWFDP	RASQSISSYLN	AASSLQS	QQSYSTPLT
5487 HC opt	DYAMH	GISWNSGSIGYADSVKG	DIAYDYSDPGGAYRYLNWFDP	RASQSISSYLN	AASSLQS	QQSYSTPLT
5536 HC/LC pair	DYAMH	GISWNSGSIGYADSVKG	DIAYDYSDLAYRYLGWFDP	RASQSISSYLN	AATAPQDVLQS	QQSYSTPLT
5987 benchmark	GFNVSYYSIH	YIYPWNDYTYYADSVKG	SRGSYYSFDY	RASQDVNTAVA	SASFLYS	QQSSVEPWT

Supplementary Figure 3.

Expression and KRAS G12V 9mer binding for TCR and CAR constructs. Transiently transfected Jurkat cells were stained with (**A**) ProL and mTCRβ for total construct expression or (**B**) pMHC tetramers either separately (2 left panels) or concomitantly (2 right panels) for binding capacity.

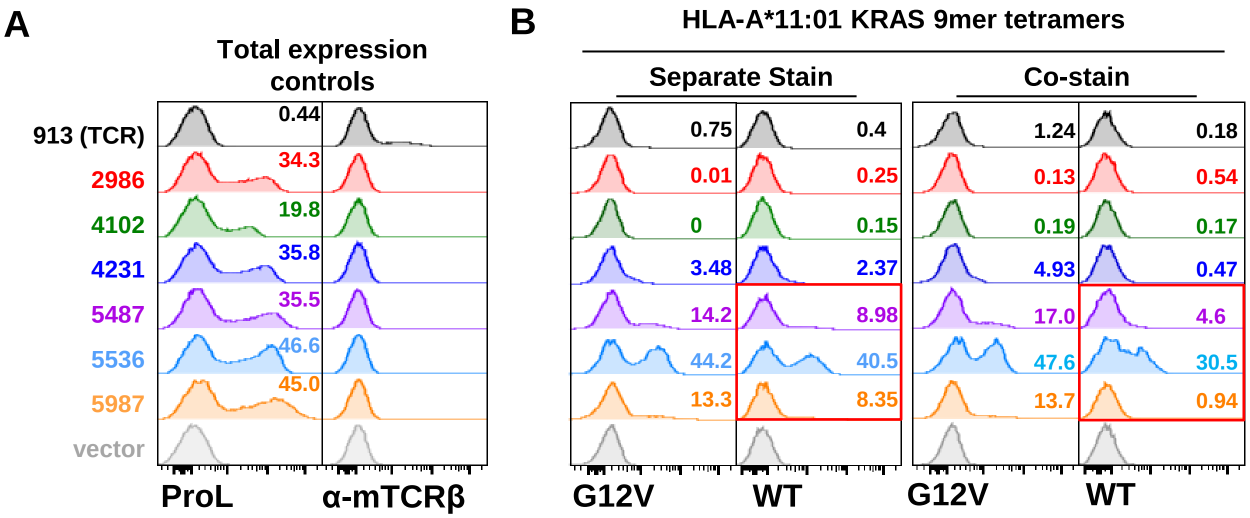

Supplementary Figure 4.

Identification of KRAS-peptide-related sequences from the human proteome for Figure 2C. (**A**) Flow schema to identify peptides. (**B**) Sequences of near off-target selectivity peptides. RNA expression levels from CCLE are shown for COLO 668 cells. NetMHC 4.0 (http://www.cbs.dtu.dk/services/NetMHC/) was used to predict display of the off-target peptides by HLA-A*11:01. na = not available.

A

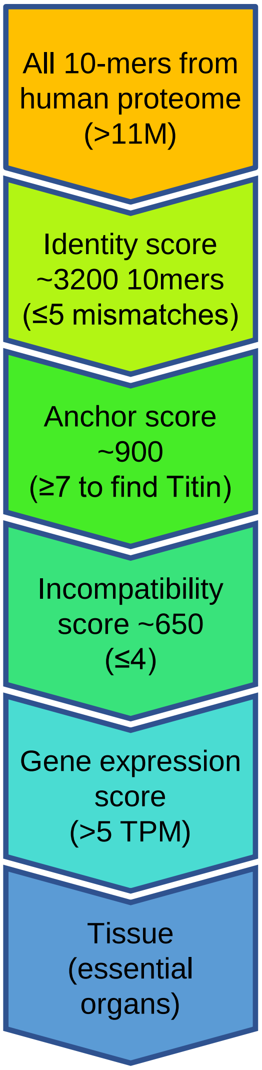

B

ID	Human gene	Peptide sequence	RNA expression (RPKM)	Peptide display prediction		
				Affinity (nM)	%Rank	Bind Level
0	KRAS G12V	VVVGAVGVGK	3.8	137.3	0.9	WB
1	TRABD	VVVGVVGMGH	19.6	14465.6	12	
2	SDHA	VVVGAGGAGL	99.7	33209.3	41	
3	PURA1	VAVKWVGVGK	na	266.5	1.3	WB
4	MPP7	VLVGPVGVGL	4.1	30219.6	33	
5	RHBT2_RHBT1	VVVGDNAVGK	na	265.9	1.3	WB
6	OTUD5	GVGGAVGVGG	18.4	38494.7	65	
7	RSLBB	AVVGASGVGK	na	161.9	0.9	WB
8	DIRA3	VVVGTAGVGK	na	274.3	1.3	WB
9	TANC1	VVVGNVGFGK	2.5	67.4	0.5	SB
10	RRAS	VVVGGGGVGK	0.8	528.3	1.9	WB
11	RASM	VVVGDGGVGK	na	329	1.4	WB
12	MIRO2	KVVGARGVGK	na	320.1	1.4	WB
13	DIRA1	VVFGAGGVGK	na	69.7	0.5	SB
14	RIT1	VMLGAGGVGK	5.2	213.6	1.1	WB
15	MRP6	AVVGPVGAGK	na	135.7	0.8	WB
16	RHES	VVLGASRVGK	na	98	0.7	WB
17	RAP2C	VVLGSGGVGK	10.7	168.6	1	WB
18	RAB36	VVVGDLYVGK	6.2	97.6	0.7	WB
19	MRP3	AVVGPVGCGK	na	145.7	0.9	WB
20	MRP1	AVVGQVGCGK	na	319.4	1.4	WB
21	DIRA2	AVFGAGGVGK	na	57.5	0.4	SB
22	CRNS1	LVVGAGGVSK	na	227.9	1.2	WB
23	RHOQ	VVVGDGAVGK	2.5	262	1.3	WB
24	RAB23	VVVGNGAVGK	3.5	257.6	1.2	WB

Supplementary Figure 5.

Functional tests on TAP-sufficient cell lines reveal cross reactivity correlates with HLA-A*11:01 expression. (**A**) Indicated cell lines were loaded with serially-diluted G12V 10mer peptide overnight and subsequently co-cultured for 6 hours with Jurkat-NFAT-luciferase cells transiently transfected with indicated CARs. (**B**) HLA-A*11 antibody staining on indicated cell lines.

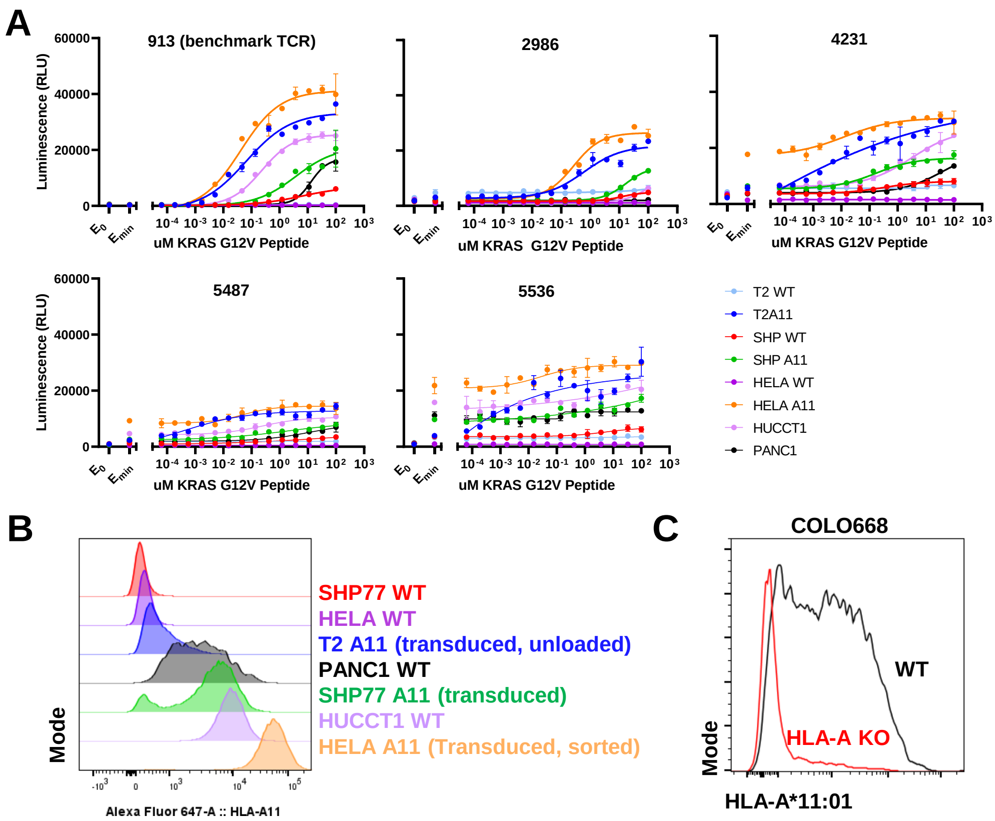

Supplement: Supplementary Data — S.Figures 1-5 and S.Table 1 [file crc-21-0165-s01.pdf]
